# Active optical boundary recognition with boron powder injection in a magnetic confinement device

**DOI:** 10.1038/s41598-026-37469-z

**Published:** 2026-01-27

**Authors:** Dong Guo, Yuejiang Shi, Qifeng Xie, Hongyue Li, Chao Wu, Jia Li, Tongqing Zhou, Yonghua Ding

**Affiliations:** 1 International Joint Research Laboratory of Magnetic Confinement Fusion and Plasma Physics, State Key Laboratory of Advanced Electromagnetic Engineering and Technology, School of Electrical and Electronic Engineering, Huazhong University of Science and Technology, Wuhan, 430074 People’s Republic of China; 2 ENN Science and Technology Development Co., Ltd., Langfang, 065001 China

**Keywords:** Engineering, Materials science, Optics and photonics, Physics

## Abstract

Accurate determination of the plasma boundary—especially the Last Closed Flux Surface (LCFS)—is crucial for understanding equilibrium, optimizing confinement, and achieving effective control in magnetic confinement devices. Conventional detection methods, such as magnetic reconstructions (e.g., EFIT) and passive optical diagnostics, have limitations during transient or low-emission conditions. We present an innovative plasma boundary identification technique that uses boron powder injection in the EXL-50U spherical torus device. When the boron particles ablate at the plasma boundary, they emit a strong localized line radiation of boron in the visible spectral range, serving as a clear, real-time marker for LCFS detection. By combining precise camera calibration with boron-filter-based contrast enhancement, our method provides high spatial accuracy and fast response with minimal hardware complexity. We describe the principles of the technique, experimental setup, and data processing workflow, and compare its performance to established boundary detection methods. Lastly, we explore the potential of active boron injection for advanced plasma control in future high-performance fusion devices, emphasizing its ability to meet the demanding requirements of long-pulse and reactor-grade plasmas.

**Supplementary Information:**

The online version contains supplementary material available at 10.1038/s41598-026-37469-z.

## Introduction

Nuclear fusion is considered one of the most promising long-term options for sustainable energy, with the tokamak design remaining the most advanced route toward reactor-grade plasmas. A crucial element of tokamak operation is the precise identification of the plasma boundary, particularly the LCFS, which governs equilibrium reconstruction, confinement optimization, and active plasma control. As devices progress toward long-pulse, high-power operation such as ITER and DEMO^1,2^, the requirements for boundary diagnostics have become increasingly strict, demanding real-time, high-precision, and model-independent methods^3–5^.

Conventional boundary reconstruction methods, such as EFIT, rely on magnetic diagnostics to indirectly infer the plasma shape^6,7^. As fusion research advances toward long-pulse and steady-state operation, these measurements, which rely on electronic integrators, are susceptible to signal drift over prolonged periods, causing accumulating errors^8,9^. And inaccuracies under transient conditions such as edge-localized modes (ELMs) or vertical displacement events (VDEs)^10^. Errors are particularly pronounced in low safety factor or non-circular plasmas, where equilibrium assumptions are less valid^11^.

Optical diagnostics have emerged as a complementary approach, offering high spatial resolution, fast response, and immunity to electromagnetic interference^12,13^. Passive boundary identification, typically through imaging hydrogen Balmer-alpha (Hα) emission, has demonstrated centimeter-level accuracy on devices such as EAST, TCV, and HL-3^14,15^. However, these methods are typically passive, relying on spontaneous edge emission that can be affected by background light, vessel reflections, and impurity transport^16^. The determined edge location does not always match the actual LCFS, particularly in plasmas with complex edge physics or low emissivity^17^.

To address these challenges, we suggest an active optical boundary detection method based on boron powder injection in the EXL-50U^18,19^ spherical torus. The main idea is to introduce a localized, temporary marker—boron particles—that emit bright visible light upon ablation at the plasma boundary^20^. By injecting boron along specific trajectories and capturing the emitted light with calibrated cameras, the position of the LCFS can be directly determined through geometric reconstruction. This method reduces dependence on emission profile assumptions and equilibrium models, providing a robust and potentially self-calibrating diagnostic for boundary detection.

Our approach builds on recent progress in real-time edge diagnostics and active impurity injection strategies. Notably, real-time boronization techniques have been studied in devices such as ITER, DIII-D, and LHD^21–23^, where controlled boron seeding enhances wall conditions and plasma performance. Additionally, research has demonstrated that boron emission lines, especially the B II doublet near 703 nm, offer a clear spectral signature suitable for optical filtering and fast photodiode detection. By integrating these insights with precise camera geometric calibration and trajectory modeling, our system delivers a spatially resolved and temporally responsive signal of the LCFS position.

In this paper, we report the first demonstration of active boundary identification with boron powder injection on the EXL-50U tokamak. We describe the experimental results, and validation against standard optical boundary methods. Finally, we discuss the advantages, limitations, and future directions of this technique, emphasizing its potential integration into plasma control systems for long-pulse discharges.

## Results

### Single-camera determination of Boron ablation and LCFS location in EXL-50U

In these experiments, boron particles were dropped vertically into the plasma from a top port at a toroidal angle of 60° in EXL-50U. Upon reaching the plasma edge, the particles ablated and produced bright, localized emission, which served as a clear marker of the LCFS.

**Fig. 1:**
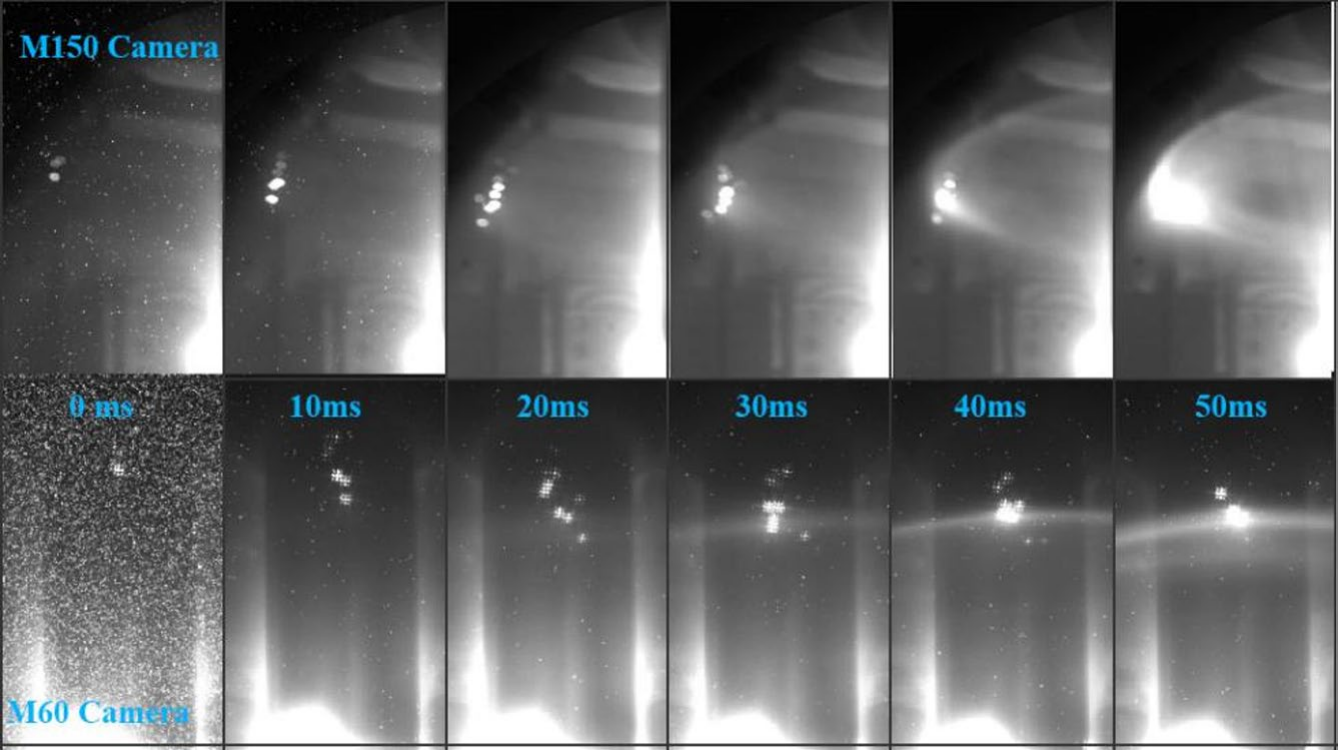
Time-lapse sequence showing boron powder ablating at the edge of the EXL-50U plasma. The images were taken simultaneously by the M150 camera (top row) and the M60 camera (bottom row). Timestamps are relative to the initial appearance of the powder in the view.

Two visible-light cameras, M150 and M60, simultaneously recorded this process from different viewing angles. Here, “M” denotes a mid-plane installation port, and the number refers to the toroidal angle of the flange. Figure 1 presents a sequence of images captured during a single injection event. A distinct phenomenon is observed: as the clusters cross the plasma boundary, their brightness increases sharply due to intense interaction with the hot plasma. Within ~ 30 ms, initially faint traces of powder evolve into luminous emission spots. These emissions provide the principal signal for our active boundary diagnostic.

As the M60 camera shares the same toroidal angle (60°) with the powder injection device, it provides a direct, head-on view of the powder’s vertical descent under gravity. The images reveal a clear free-fall phase of ~ 20 cm before the onset of ablation, confirming that the particles travel ballistically under gravity until they reach the LCFS. After crossing the boundary, the clusters cease downward motion and instead rotate toroidally along magnetic field lines. By contrast, the M150 camera, positioned at a 150° toroidal angle, depicts compressed vertical motion and reduced sensitivity to height variations. Consequently, in practice, LCFS determination relies on selecting emission clusters that appear brightest and most extended, as these indicate full ablation and definitive contact with the boundary.

**Fig. 2:**
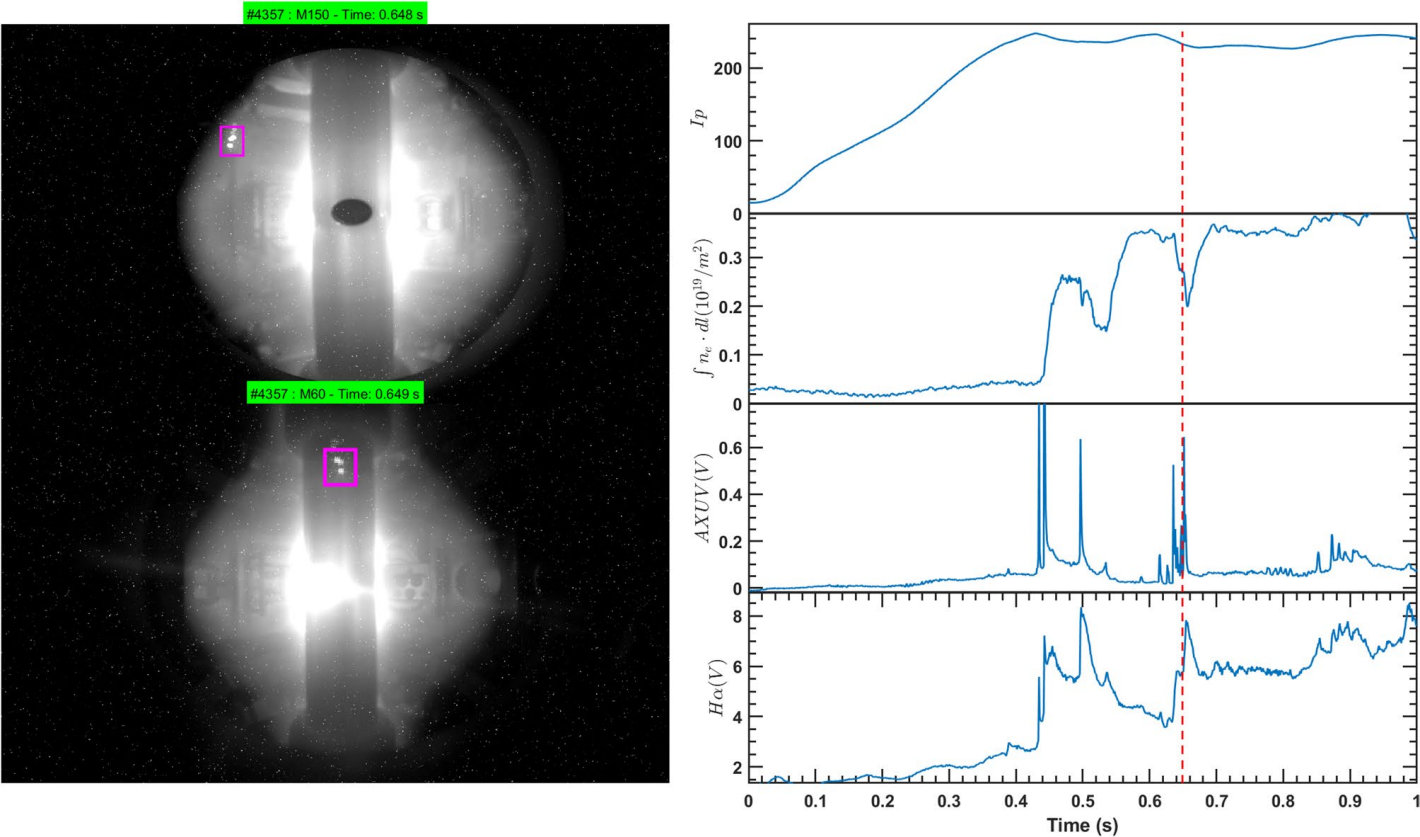
Images from two visible light cameras positioned at 150° and 60° toroidal angles show the plasma during a discharge, along with the time evolution of key plasma parameters including plasma current (Ip), line-integrated electron density (ne), AXUV radiation, and Hα radiation. The magenta box highlights the boron powder clusters, marked by an increase in emission as the boron powder begins to ablate, producing bright clusters in the images.

The images in Fig. 2 present plasma observations from the M150 and M60 cameras together with the temporal evolution of key parameters, including plasma current (Ip), line-integrated electron density (ne), AXUV radiation, and Hα emission. At 0.62 s, when boron powder is injected from the top of the vacuum chamber, the particles first follow a gravitational trajectory before interacting with the plasma edge. Their contact with the LCFS coincides with a rise in AXUV and Hα signals, indicating enhanced radiation and recycling processes triggered by ablation. On the left of the figure, two distinct boron clusters are visible in the camera images, with consistent detection from both M150 and M60 perspectives.

### Validation: comparison with conventional optical boundary

Convectional optical boundary reconstruction uses the Balmer-alpha (Hα) emission, which forms a thin, bright layer that peaks sharply at the LCFS. The unique plasma conditions required for Hα excitation make it a reliable marker for identifying the plasma boundary.

**Fig. 3:**
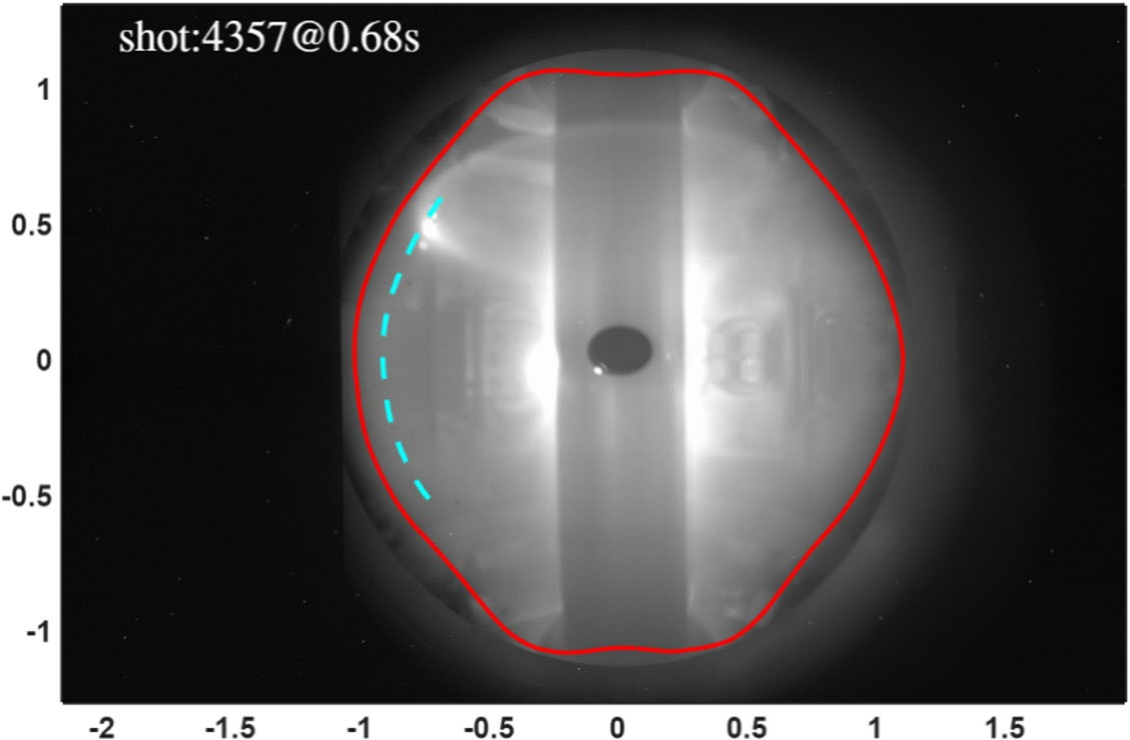
#4537 camera (M150) optical boundary recognition results; the red line shows the approximate LCFS position obtained by threshold segmentation, and the cyan indicates the result obtained through coordinate transformation.

The LCFS is identified by applying a brightness threshold to 2D camera images, which segments the bright core plasma from the darker scrape-off layer (red line, Fig. 3). The boundary pixels from the image are then mapped to physical (R, Z) coordinates using a calibrated camera model and a coordinate transformation to correct for 3D perspective effects (cyan dashed line)^3,24^. While this method is accurate in the high-contrast mid-plane region, it fails near the divertors. In these areas, strong background radiation from intrinsic impurities drastically reduces the brightness contrast, leading to large errors in the identified LCFS position.

**Fig. 4:**
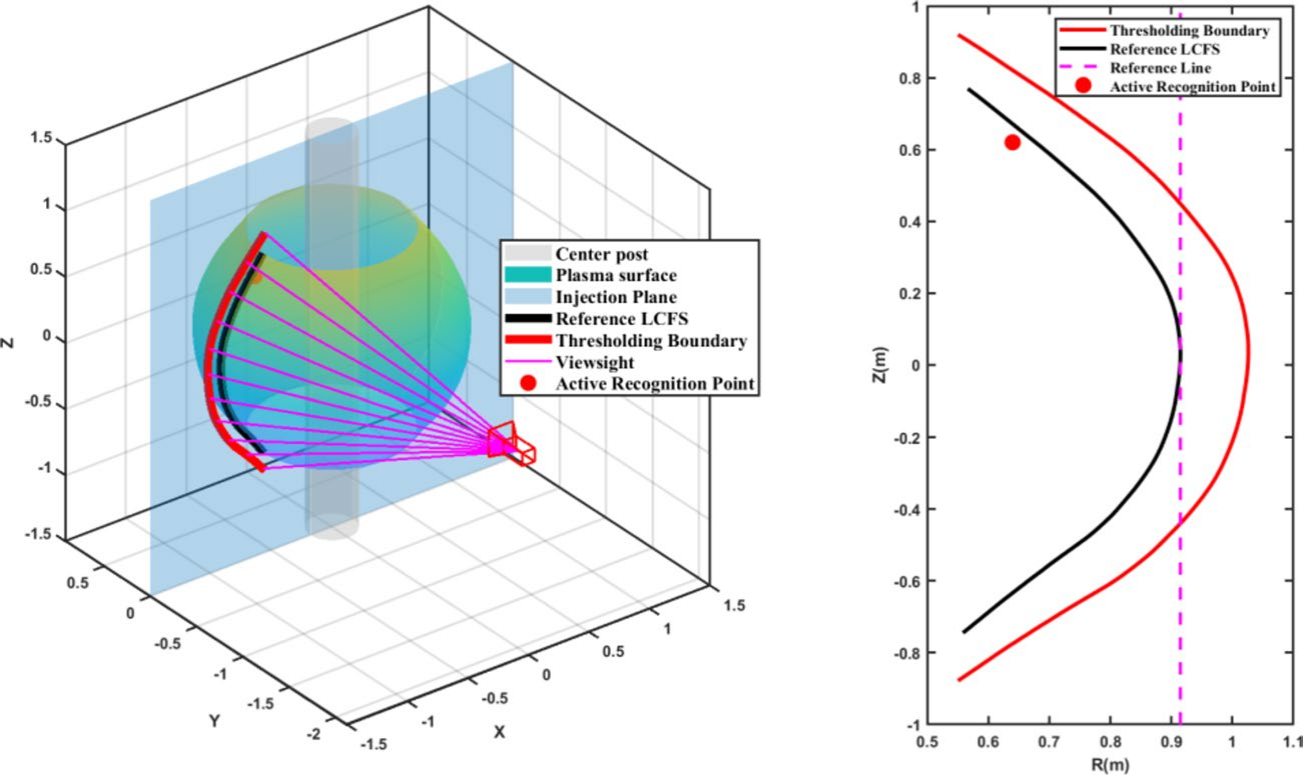
(Left) 3D schematic of the single-camera setup, with the red dot marking the LCFS identified by the active method. (Right) Comparison of LCFS reconstructions on the poloidal plane: black, traditional thresholding with geometric correction; red, thresholding before correction; red dot, active detection result.

We constructed a virtual 3D model to illustrate the geometric relationships between the vacuum vessel, the plasma, the optical boundaries, the coordinate transformations, and the camera model. As shown in Fig. 4 left, the center post (gray cylinder) represents the central column, while the plasma surface (blue-green) depicts the main plasma volume. The projection plane (blue rectangle) is positioned at a toroidal angle of 60° and aligns with the injection path of the boron particles. The camera is situated at 150°, creating a 90° viewing angle between its optical axis and the projection plane. Pink rays illustrate the lines of sight from the calibrated camera toward the plasma boundary, and the red markers indicate boron ablation sites. On the right side of Fig. 4, the red line shows the plasma boundary before coordinate transformation, while the black line displays the corrected LCFS after the transformation.

**Fig. 5:**
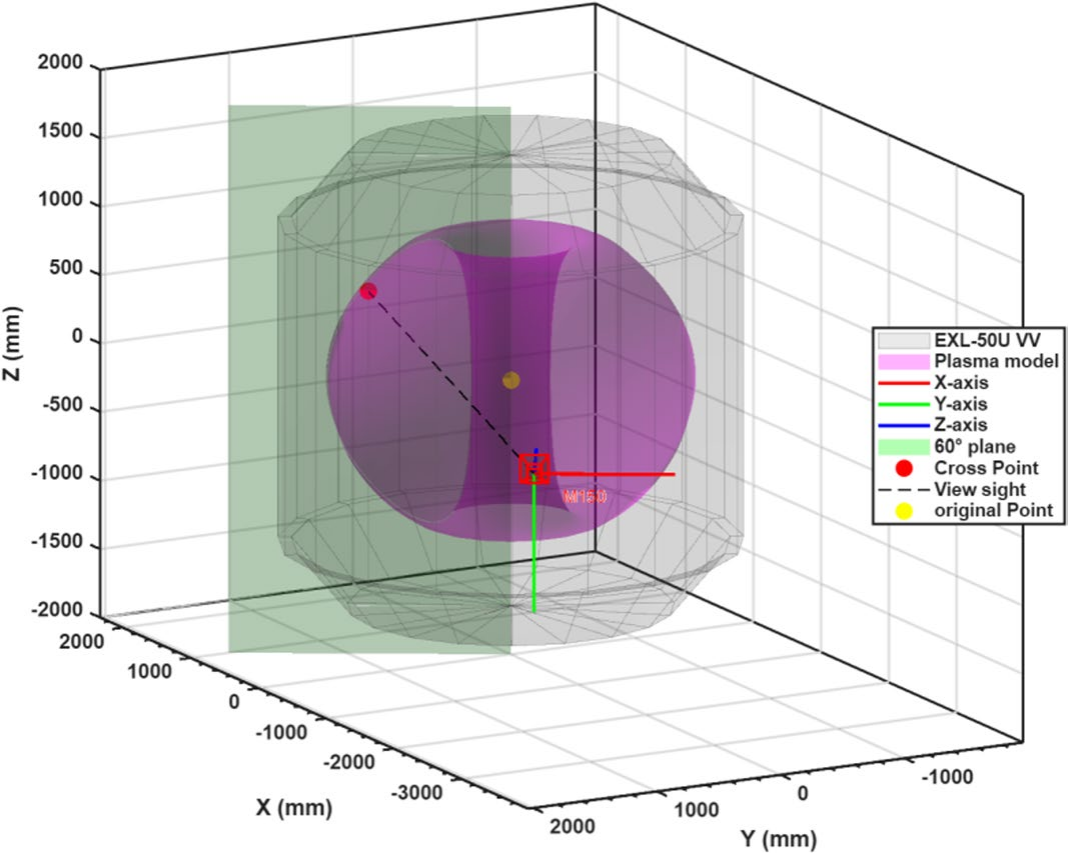
Illustration of the geometric relationships in the plasma optical boundary identification method during boron powder injection conditions.

The second method, introduced in this work, uses boron injection as an active “marker”^23,25^. First, we corrected lens distortion using the determined intrinsic parameters^26^. Next, we manually selected the intense emission spot corresponding to the boron ablation. From the camera’s optical center, we projected a ray through this emission point. Because the boron was dropped at the 60° toroidal position, a plane was defined in that toroidal sector, and the intersection of the plane with the ray provided the exact three-dimensional location of the boron ablation in the vacuum vessel. This geometric method depends only on camera calibration and injection geometry, minimizing assumptions related to plasma edge physics. Figure 5 illustrates the geometric relationships involved in the plasma optical boundary identification method during boron powder injection. The green color shows the plane where the boron powder injection angle is located. The gray EXL-50U device represents the vacuum chamber. Purple indicates the plasma geometry at the R = 700 mm position. The black dashed line connects the camera’s optical center with the boron ablation point. The intersection of the green plane and this connection line mark the absolute LCFS position. The red, green, and blue lines show the X, Y, and Z axes of the camera’s local reference frame, respectively.

Comparison of the reconstructed LCFS between these two methods shows good agreement. For example, Fig. 4 (right panel) demonstrates this comparison. The plasma boundary obtained through the traditional threshold segmentation method (after full geometric transformation) is shown as the black line. The local boundary position of the plasma, determined using our new active recognition method with boron powder, is represented by the red dot. The red dot’s position aligns closely with the black line, confirming the validity of our new local measurement technique. The minor deviation of approximately 4 cm in the divertor region is expected, as this area involves more complex image information and less precise global thresholding methods.

The experimental results indicate that active boron powder injection can still offer a more precise reference point for the LCFS under complex optical conditions, such as near the divertors, because of its “active marker” property. This complements the conventional method of identifying the LCFS boundary through thresholding segmentation. Additionally, if the active marking points are strategically arranged along the entire LCFS path after proper design and planning, the whole LCFS path can be determined through fitting. Furthermore, this approach is real-time and requires minimal computation, making it well-suited for deployment in real-time boundary control systems to manage long-pulse discharges.

### Design of an active boron powder injection diagnostic system for plasma optical boundary recognition

**Fig. 6:**
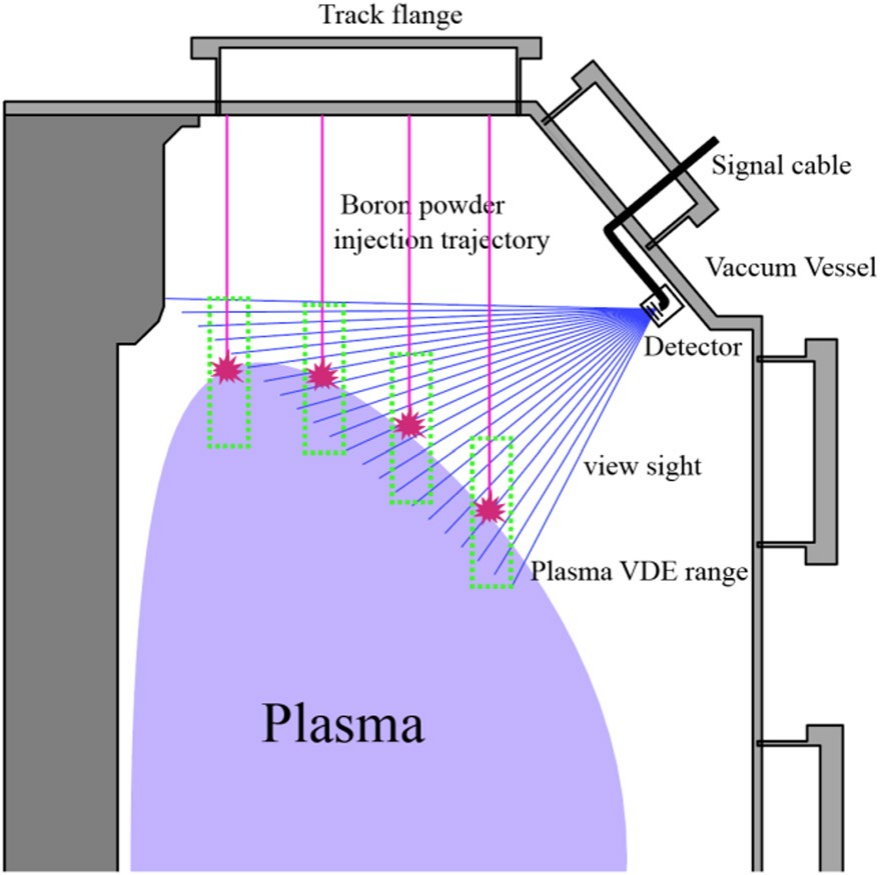
Schematic of the diagnostic system for active boron powder injection. The U-shaped injection flange, particle trajectories, and ablation points near the plasma boundary are illustrated. The detector arrangement with a narrowband 703 nm filter is shown, used to record emission from boron ablation for boundary identification.

A dedicated diagnostic system was constructed to enable active recognition of the plasma boundary using boron powder injection and optical emission detection. As illustrated in Fig. 6, powder is released from several injectors mounted along a U-shaped flange at the vessel top, providing coverage across the poloidal cross-section. Under gravity, particles enter the edge plasma and ablate upon contacting the LCFS, producing localized B II emission near 703 nm that serves as a direct boundary marker.

Detection is performed with an Avalanche Photodiode (APD) array located on the weak-field side, aligned with the injection plane. Because APDs respond broadly to visible light, background plasma radiation can overwhelm the signal. A narrowband interference filter centered at 703 nm is therefore installed, isolating the boron line while suppressing out-of-band light. This selective filtering markedly improves the signal-to-noise ratio and prevents detector saturation, allowing stable, linear measurements at sampling rates up to 100 kHz.

Emission localization is achieved by identifying the intensity peak along each detector line-of-sight and intersecting it with the known trajectory of the injected particle stream. Repeating this calculation across multiple injection heads and time frames yields a 3D reconstruction of the evolving boundary. The method combines hardware simplicity with modest computational demands, enabling real-time analysis. Its efficiency and clarity make it a practical candidate for integration into feedback control systems in future fusion devices.

## Discussion

We have demonstrated an active diagnostic approach for identifying the Last Closed Flux Surface (LCFS) in a spherical torus through boron powder injection. On EXL-50U, particle ablation at the edge produced localized emission that served as a temporary boundary marker. These signals coincided with conventional optical reconstructions, confirming accuracy in the midplane region, while providing enhanced clarity near the divertor where strong background radiation often limits passive imaging. The results highlight that active edge marking can directly complement traditional diagnostics by supplying clear geometric references in optically complex environments.

A key strength of the method is its independence from equilibrium reconstruction. Tools such as EFIT rely on axisymmetric equilibrium assumptions, and passive line-of-sight measurements are prone to uncertainties linked to background light and emission overlap. In contrast, boron ablation produces discrete, geometrically resolvable signals with reduced ambiguity. Nonetheless, important limitations remain. The spatial resolution is constrained by the extended emission of ablation clusters, and the technique is currently restricted to a single poloidal view. The ablation process depends on local edge conditions, leading to variability in emission strength and position, and small trajectory deviations may affect boundary reconstruction. Impurity accumulation is another factor: while injection rates of 2–6 mg/s provided reliable signals, they temporarily increased boron ion density to ~ 20% of the hydrogen background, underscoring the need for impurity management in long pulses or reactor-scale scenarios.

Future improvements may significantly expand the diagnostic’s scope. Multi-camera triangulation could support full 3D reconstruction, while fast detectors would allow real-time feedback useful for plasma control, including heat flux mitigation and stability management. With further integration of machine-learning algorithms to classify signals under fluctuating edge conditions, this active approach offers a promising pathway toward precise, real-time plasma boundary identification in next-generation devices.

## Methods

### Boron powder injection system

**Fig. 7:**
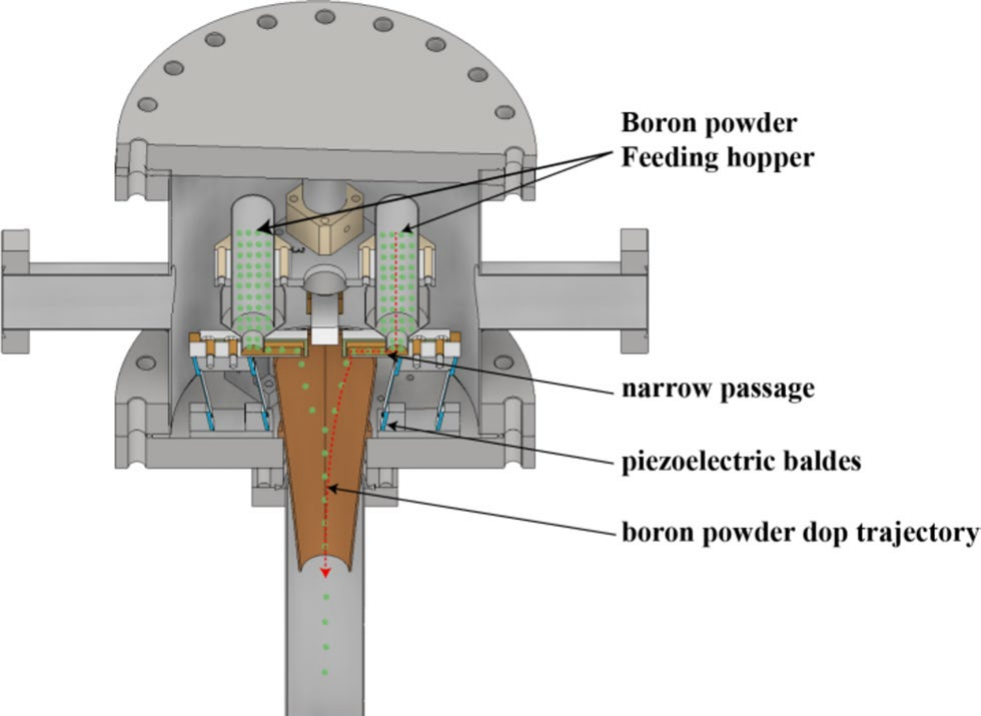
Schematic of the boron powder injector used for active optical boundary detection in the EXL-50U spherical torus.

The boron injection system is mounted at a top port with a toroidal angle of 60° and comprises four independently controlled hoppers, of which two are currently operational. Each hopper connects vertically to a conical delivery tube via a narrow channel, enabling precise powder transport. The feed rate is regulated by piezoelectric blades that modulate vibration frequency and amplitude, establishing a calibrated relation between control parameters and injection rate. Boron powder of ~ 150 μm particle size and 99.9% purity can be delivered at 1–10 mg/s for durations from 50 ms to several seconds (Fig. 7). As the particles fall under gravity and interact with the plasma edge, ablation occurs, producing visible B II emission near 703 nm that marks the LCFS.

**Fig. 8:**
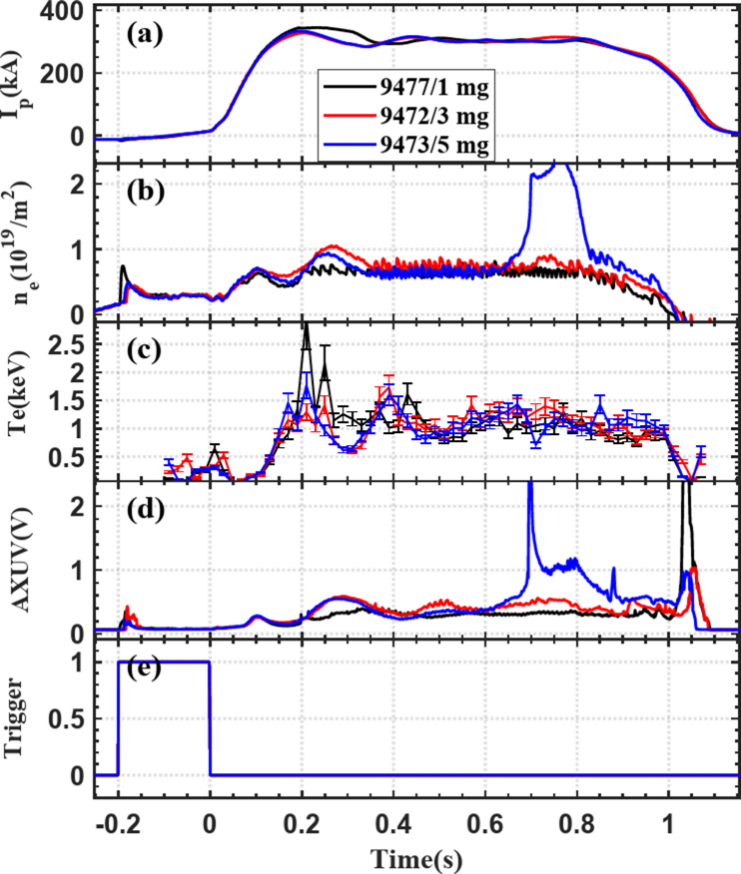
Temporal evolution of plasma parameters in EXL-50U with different boron injection amounts: 1 mg (black), 3 mg (red), and 5 mg (blue). The plots show the temporal evolution of plasma current, line-integrated electron density, core temperature, total radiation, and the trigger signal associated with powder injection.

To evaluate the diagnostic’s impact, discharges with comparable initial conditions were performed with different injection doses (1, 3, and 5 mg), as shown in Fig. 8. For 1 mg and 3 mg cases, key plasma parameters—including plasma current (Ip), line-integrated density (ne), core electron temperature (Te), and total radiation—remained essentially unchanged, indicating negligible disturbance. At 5 mg, a transient rise in edge density and radiation was observed around 0.65 s, consistent with the arrival of particles at the boundary, but confinement metrics such as Ip and Te remained largely unaffected. These results confirm that controlled injection rates, typically below ~ 3 mg/s, allow effective boundary detection with minimal impact on core plasma performance.

### Diagnostic

**Fig. 9:**
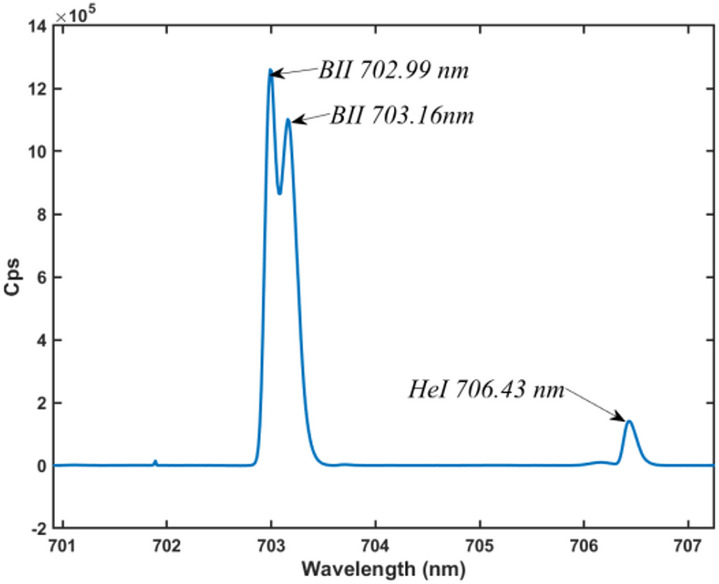
Visible-light spectrum recorded by a Princeton Instruments SCT320 spectrometer during boron powder injection experiments.

a Princeton Instruments SCT320 spectrometer was used to analyze the visible emission spectrum during boron powder injection experiments. As shown in Fig. 9, two prominent emission peaks were observed at 702.99 nm and 703.16 nm, corresponding to characteristic B II transitions listed in atomic databases. A comparatively weaker He I line was also detected at 706.43 nm. This spectral separation confirms that the 703 nm band is well-suited for isolating boron-specific emission, enabling the use of narrowband filters to improve detection contrast and suppress background light.

## Optical imaging and camera geometric calibration

Accurate reconstruction of ablation positions requires precise geometric calibration of each camera. The procedure was performed in two stages: intrinsic calibration to correct lens properties, and extrinsic calibration to align the camera with the coordinate system of the tokamak.

*Intrinsic calibration.* Focal length, principal point, and lens distortion were obtained with Zhang’s checkerboard method. A calibration grid of known size was imaged at multiple orientations, and the pixel coordinates of the corners were matched to their known 3D positions using MATLAB’s Camera Calibrator toolbox. This produced the intrinsic matrix for distortion correction and projection.

**Fig. 10:**
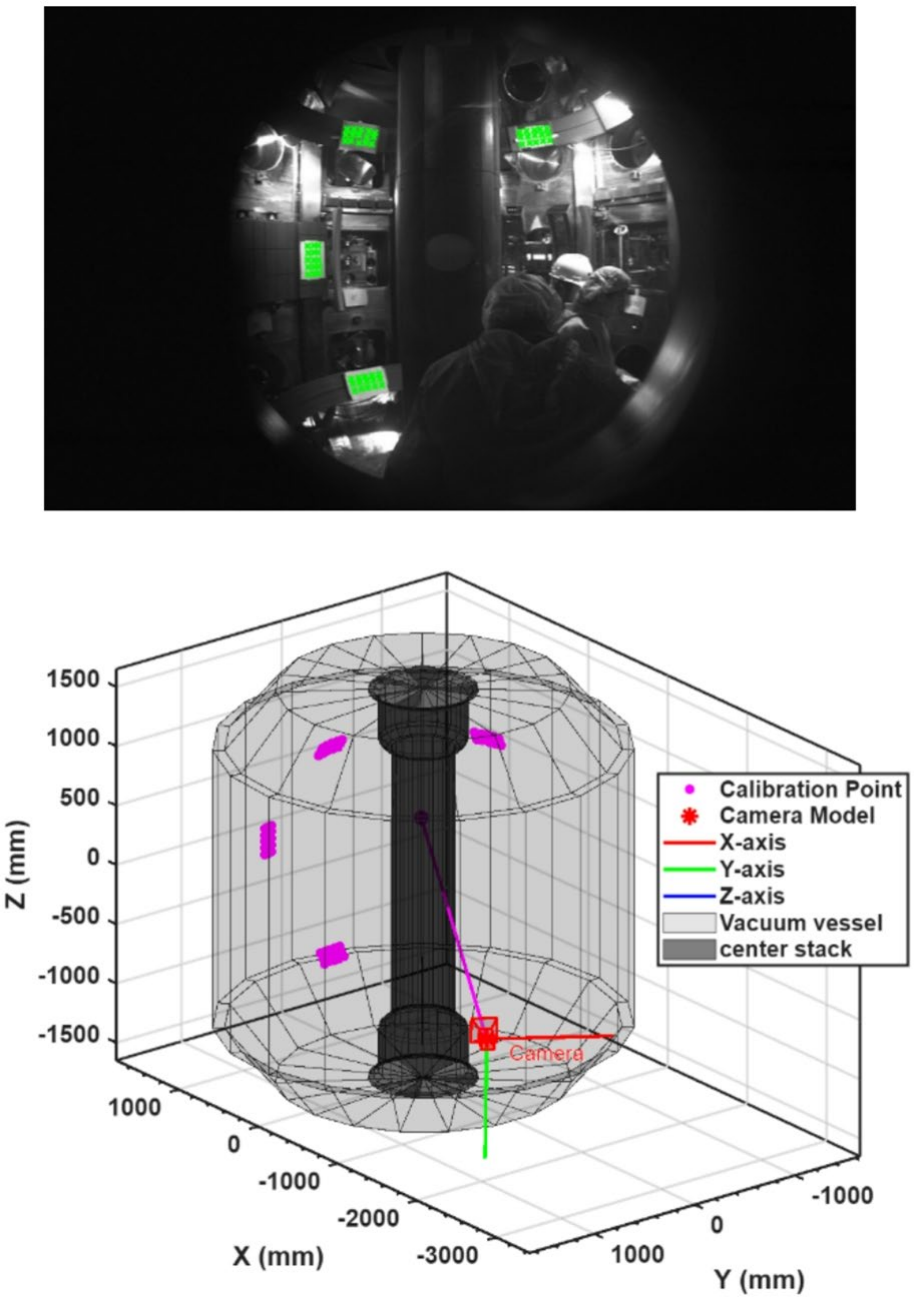
(**a**) In-vessel image showing reference markers used for camera calibration. (**b**) 3D model of the EXL-50 vacuum vessel with the corresponding reference points (magenta) and coordinate axes (RGB), used to align the camera geometry with the device.

*Extrinsic calibration.* After lens effects were corrected, the spatial relation between camera and device coordinates was determined. As shown in Figs. 10(a) and (b), Reference markers were installed at measured positions inside the vessel using a laser tracker and subsequently imaged. Matching their known 3D locations with the corresponding image pixels provided the camera rotation and translation parameters. Calibration accuracy was assessed by reprojecting the 3D reference points onto the image plane; deviations were limited to a few pixels. A 3D visualization of the camera frustum further confirmed consistency between the optical model and the installed geometry.

## Supplementary Information

Below is the link to the electronic supplementary material.

Supplementary Material 1

## Data Availability

The datasets generated and/or analyzed during the current study are available from the corresponding author on reasonable request.
