# Supplementary figures and images for: Active optical boundary recognition with boron powder injection in a magnetic confinement device
Source: Sci Rep. 2026 Jan 27;16:6326. doi: 10.1038/s41598-026-37469-z (PMC12905401; doi:10.1038/s41598-026-37469-z)

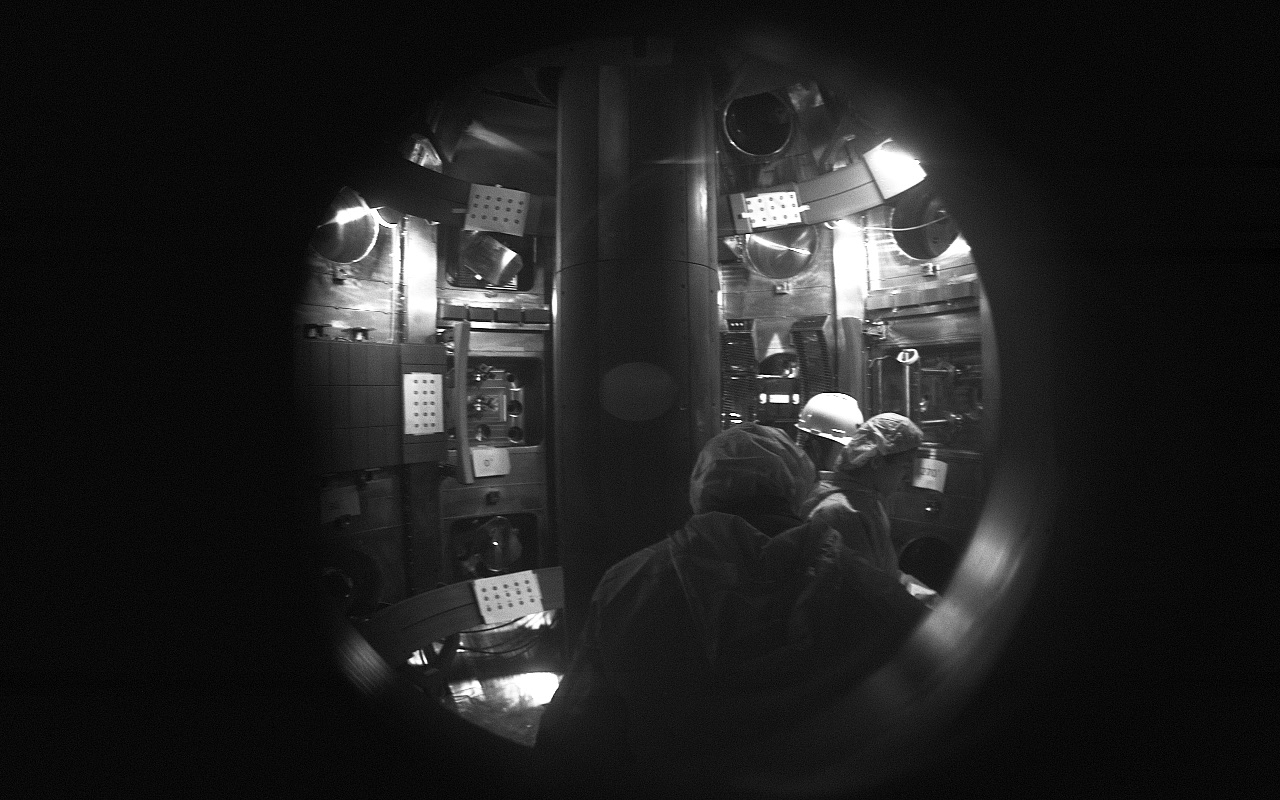

Supplement: Supplementary file 1 — Supplementary Material 1 [file 41598_2026_37469_MOESM1_ESM.zip › figure 10(a) raw image.jpg]

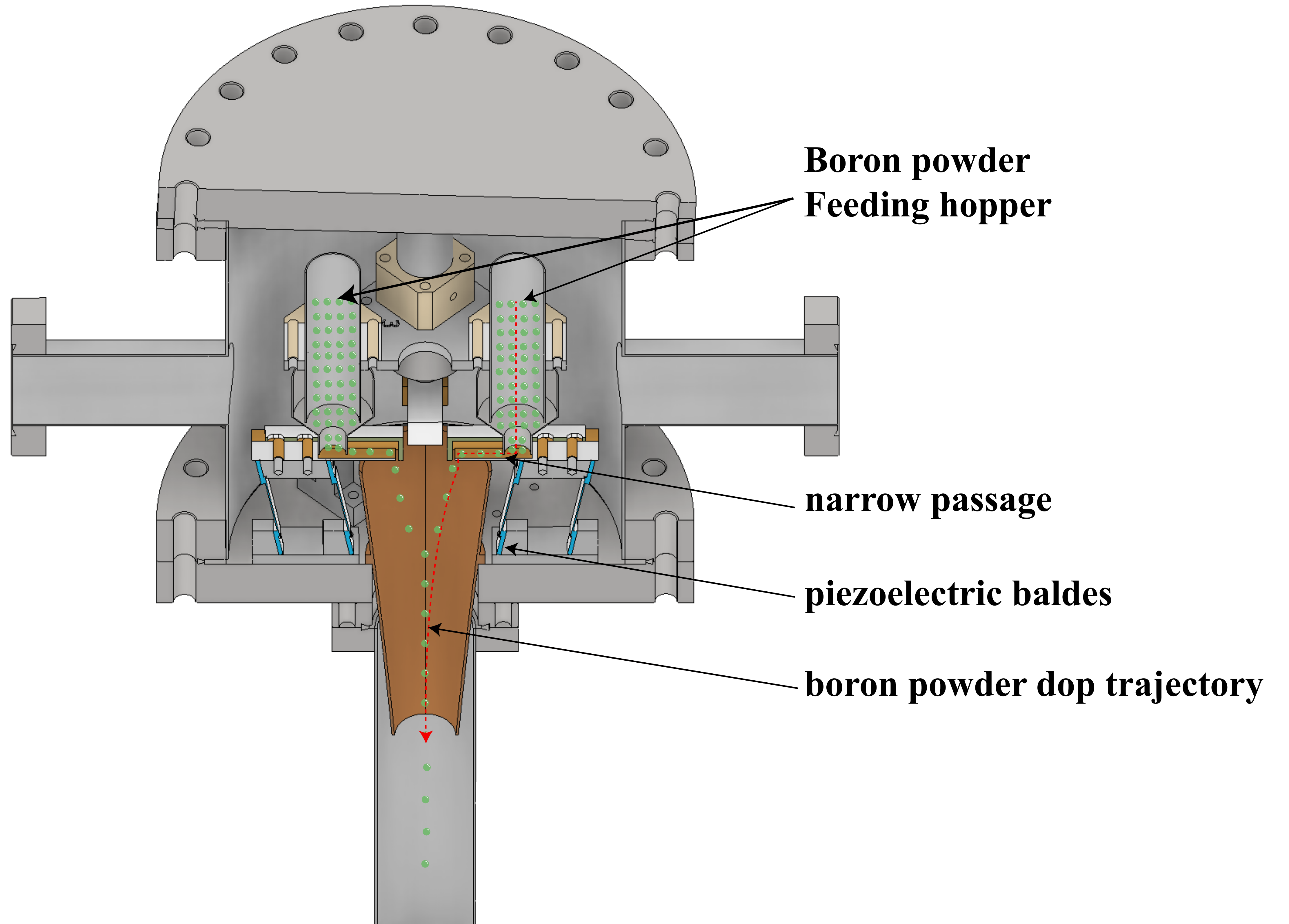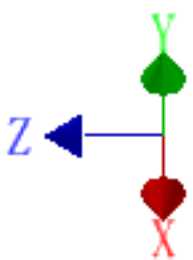

Supplement: Supplementary file 1 — Supplementary Material 1 [file 41598_2026_37469_MOESM1_ESM.zip › figure7.pdf]
